# A Novel Assessment Tool for Impulsive Aggression in Children with Attention-Deficit/Hyperactivity Disorder

**DOI:** 10.1089/cap.2019.0035

**Published:** 2019-10-07

**Authors:** Gianpiera Ceresoli-Borroni, Tesfaye Liranso, Scott T. Brittain, Daniel F. Connor, Christopher J. Evans, Robert L. Findling, Steve Hwang, Shawn A. Candler, Adelaide S. Robb, Azmi Nasser, Stefan Schwabe

**Affiliations:** ^1^ Department of Clinical Research, Supernus Pharmaceuticals, Inc., Rockville, Maryland.; ^2^ Department of Psychiatry, Division of Child and Adolescent Psychiatry, University of Connecticut School of Medicine, Farmington, Connecticut.; ^3^ Department of Research Science, Endpoint Outcomes, Boston, Massachusetts.; ^4^ Department of Psychiatry and Behavioral Sciences, The Johns Hopkins University, Baltimore, Maryland.; ^5^ Department of Psychiatry and Behavioral Sciences, Kennedy Krieger Institute, Baltimore, Maryland.; ^6^ Department of Medical Affairs, Supernus Pharmaceuticals, Inc., Rockville, Maryland.; ^7^ Department of Psychiatry and Behavioral Sciences, Children's National Medical Center, Washington, District of Columbia.; ^8^ Department of Psychiatry and Behavioral Sciences, George Washington University, Washington, District of Columbia.; ^9^ Department of Research and Development, Supernus Pharmaceuticals, Inc., Rockville, Maryland.

**Keywords:** impulsive aggression diary, assessment tool, attention-deficit/hyperactivity disorder, aggression, psychometrics

## Abstract

***Objective:*** To establish the validity and reliability of a provisional 30-item impulsive aggression (IA) diary in children (ages 6–12 years, inclusive) with attention-deficit/hyperactivity disorder (ADHD).

***Methods:*** The provisional 30-item IA diary was administered for 14 days to parents of children with ADHD and IA symptoms (*n* = 103). Key inclusion criteria: confirmed ADHD diagnosis; signs of IA as measured by a Retrospective-Modified Overt Aggression Scale (R-MOAS) score ≥20 and an Aggression Questionnaire score of −2 to −5. Analyses included inter-item correlations, exploratory factor analysis (EFA), item response theory (IRT) modeling, internal consistency, test–retest reliability (TRT), concurrent validity (estimated by correlation between the IA diary and the R-MOAS/Nisonger Child Behavior Rating Form), and known-groups methods.

***Results:*** The prevalence rates of 15 (50.0%) items were found to be too low (<1%) for analysis; three items with prevalence rates ≤1% were retained, as content validity was deemed high by clinical experts. The remaining 12 behavior items had prevalence rates of 2.7%–73.6%. EFA and IRT models confirmed two subdomains in the IA diary included within a general domain of IA behavior frequency, yielding a single total behavioral frequency score (TBFS). Internal consistency was high for this TBFS (marginal reliability = 0.86 and α = 0.73). TRT for the TBFS, based on the intraclass correlation coefficient, was 0.8. Concurrent validity of TBFS with R-MOAS ranged from *r* = 0.49 to *r* = 0.62.

***Conclusion:*** The final 15-item IA diary is a reliable, psychometrically validated IA measurement tool that will allow clinicians and researchers to assess the frequency of IA behavior.

## Introduction

Maladaptive aggression in children is challenging to diagnose and treat (Connor 2016). Impulsive aggression (IA) is the most common clinically aggressive behavior in children, with an occurrence rate of ∼80% within aggressive children (Barratt et al. 1999; Blader et al. 2009; American Psychiatric Association 2013). Associated with various neuropsychiatric disorders, IA can be characterized as a reactive, overt, and maladaptive form of aggression occurring outside the acceptable social context. IA can be physical or verbal; it is retaliatory and lacks a discernable goal (Jensen et al. 2007; Connor 2016; Miles et al. 2016; Saylor and Amann 2016). The impetuous/explosive nature of IA distinguishes this aggregate set of behaviors from other conduct disorders such as disruptive mood dysregulation disorder, which manifests as chronic irritability that may lead to aggressive behavior (Barratt et al. 1999; American Psychiatric Association 2013; Connor 2016; Gurnani et al. 2016).

IA symptoms are a major clinical concern, amplifying the risk of poor social outcomes and functional difficulties in those with comorbid disorders, such as attention-deficit/hyperactivity disorder (ADHD) (Jensen et al. 2007; Saylor and Amann 2016). Approximately 26% of children receiving medication for ADHD have persistent IA symptoms. Recent findings indicate that functional impairment associated with ADHD may be more attributable to aggression or irritability than to core ADHD symptoms themselves (Saylor and Amann 2016), underscoring the need to identify and effectively treat aggression.

Some components of IA behavior (e.g., shouting or injuring self/others) can be monitored using existing instruments, including the Retrospective-Modified Overt Aggression Scale (R-MOAS), the Aggression Questionnaire (Vitiello et al. 1990), and the Young Mania Rating Scale (Vitiello et al. 1990; Jensen et al. 2007; Blader et al. 2009; Kaat et al. 2015). However, these scales were not developed specifically to assess IA and can capture elements of other disorders (e.g., oppositional defiant disorder, conduct disorder, and bipolar disorder) (Vitiello et al. 1990; Jensen et al. 2007; Blader et al. 2009; Kaat et al. 2015). The lack of an instrument to assess IA behavior specifically in clinical practice creates a substantial gap in our ability to effectively understand and monitor IA. Furthermore, the lack of such an established outcome measurement tool in clinical trials impairs our ability to monitor IA responsiveness to investigational treatments.

In this study, we report the development and validation of the IA diary, a novel, electronic observer-reported outcome, parent/caregiver-completed measurement tool to assess IA behavior in children (aged 6–12 years) with ADHD consistent with the Food and Drug Administration's Guidance for Industry, “Patient-Reported Outcome Measures: Use in Medical Product Development to Support Labeling Claims” (U.S. Department of Health and Human Services Food and Drug Administration 2009).

## Methods

### IA diary

A provisional diary was used in developing the IA diary described in the current study, including the same checklist of 30 single-item actions (behaviors; see Supplementary Table S1). The present study evaluates the psychometric validity and reliability of the diary as a novel IA assessment tool for children with ADHD.

**FIG. 1.**
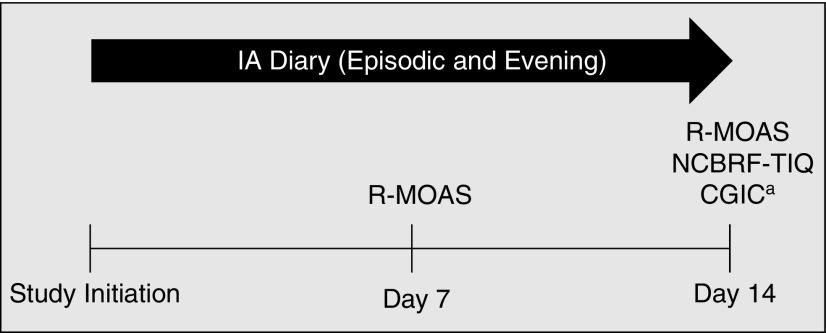
Assessment schedule. ^a^This week compared to last. CGIC, Caregiver Global Impression of Change; IA, impulsive aggression; NCBRF-TIQ, Nisonger Child Behavior Rating Form-Typical IQ; R-MOAS, Retrospective-Modified Overt Aggression Scale.

Diary items can be grouped into three hypothesized domains: verbal aggression, physical aggression directed at things or objects, and physical aggression directed at people (self or others). While some items included in the diary are also components of the R-MOAS, the IA diary uniquely consists of two parts, which parents/caregivers completed during the 14-day study. The electronic diary itself is an app-based platform (LogPad App^TM^), which was loaded onto LG Nexus 5 Android smartphones distributed to parents/caregivers during this study. The Episodic diary, completed soon after an aggressive episode occurred, captured information about the recipient of the aggressive act (e.g., hitting directed at him/herself, another person, or an animal), and how the parent/caregiver learned of the episode (e.g., directly observed or through report). The parent/caregiver completed the diary by checking all listed behaviors that applied to the episode (i.e., to indicate if the behavior occurred during the episode). The Evening diary allowed the parent/caregiver to confirm episodes recorded throughout the day and update with additional aggressive events listed in the diary that were not previously recorded. In addition to these diary components, parents/caregivers completed three independent validation assessments/measures: the Nisonger Child Behavior Rating Form-Typical IQ (NCBRF-TIQ) and Caregiver Global Impression of Change (CGIC) assessments, completed at day 14, and the R-MOAS, completed at days 7 and 14 (Fig. 1).

### Study design and participants

This was a multicenter, noninterventional, stand-alone, psychometric validation study. Eligible children were 6–12 years old and diagnosed with ADHD, as confirmed by the Diagnostic and Statistical Manual of Mental Disorders, 4th ed., Text Revision (DSM-IV-TR; American Psychiatric Association 2000 or Diagnostic and Statistical Manual of Mental Disorders, 5th ed. DSM-5; American Psychiatric Association 2013). Signs of IA behavior were required, as indicated by an R-MOAS score ≥20 and an Aggression Questionnaire (Vitiello et al. 1990) score of −2 to −5. Inclusion and exclusion criteria are summarized in Supplementary Data.

### Ethics

The study was performed in accordance with the ethical principles of the Declaration of Helsinki and consistent with Good Clinical Practice and all applicable regulatory requirements. All documentation, informed consent forms [detailing the purpose of the study, study procedure, subject(s) responsibilities, and confidentiality in the event of study publication], case report forms, and health information were approved by an institutional review board before study onset.

### Analysis samples and psychometric analyses

#### Psychometric analyses

Cross-sectional analyses (e.g., factor analysis and item response theory [IRT] modeling) were performed on data available at one sampling or one visit, including all subjects meeting inclusion criteria and completing baseline (Visit 1). Longitudinal analyses were performed on more than one sampling to evaluate subjects over time (e.g., test–retest reliability [TRT]), including all members of the cross-sectional analysis sample who completed both the baseline measure and the designated longitudinal reports.

Inter-item correlations were estimated for the final IA behavior item set. Because IA diary entries were binary in nature, tetrachoric correlations obtained from SAS's FREQ procedure were used to estimate inter-item correlations. A total of 435 inter-item correlations were possible, based on 30 potential items in the item set; therefore, a threshold of ≥0.3 was chosen (corresponding to ∼10% shared variance) to limit the number of inter-item correlations summarized. As 2 × 2 tables can degenerate for item pairs in a sample size <200, an amended inter-item correlation estimation protocol was used to circumvent design complexities associated with reporting from 2 × 2 tables. The sample of *n* = 103 used in this validation likely resulted in some degenerate 2 × 2 tables underlying the tetrachoric correlations among the 30 items, rendering correlations associated with such tables inestimable.

Data collected through the R-MOAS, the NCBRF-TIQ, the NCBRF-TIQ Disruptive Behavior (NCBRF-TIQ D-Total) scores, and the CGIC scores were also analyzed. TRT estimated the degree to which an instrument yielded similar scores at different time points using intraclass correlation coefficient (ICC, weighted scores) and Spearman rank correlations (unweighted scores). Concurrent validity defining the degree of association between two data points measured at the same time was estimated by Spearman rank correlations between the IA diary and the R-MOAS, as well as the IA diary and the NCBRF-TIQ D-Total scores.

In this IA diary validation, scores for known-groups validity, or the extent to which scores are linked to predefined health, were conditioned based on the R-MOAS clinically significant aggression categories (score of ≥24) and NCBRF-TIQ D-Total problem behavior categories (85th percentile score of ≥48), using the appropriate linear or generalized linear mixed-effect model to which the IA diary scores conform. A further description of psychometric analyses, including model fit and algorithms, is available in Supplementary Data.

## Results

### Subject characteristics

This study investigated 103 subjects from 83 households. Subjects were primarily male (70.9%), white (53.9%), and non-Hispanic (85.4%) (Table 1). Their average age was 8.7 years; most were completing the third grade at study onset. Of the participating families, 68 (81.9%) were single-child families, 11 (13.3%) were two-child families, 3 (3.6%) were three-child families, and 1 (1.2%) was a four-child family. The mean number of years since IA symptom onset was 4.9. Most children (53.4%) were seeing a psychiatrist/psychologist, approximately one-third were seeing a primary care physician (36.9%), and a few were under the care of a neurologist (9.7%). Caregivers were predominantly female (94.2%; Supplementary Table S2).

**Table 1.** Child Demographics

*Variable*	*Estimates (*N* = 103)*^a^
Child sex, *n* (%)	
Male	73 (70.9)
Female	30 (29.1)
Calculated child age, years^b^	
Mean (SD)	8.7 (2.4)
Min–Max	6–12
Hispanic, *n* (%)	
No	88 (85.4)
Yes	15 (14.6)
Race, *n* (%)^c^	
Black	29 (28.4)
White	55 (53.9)
Biracial	18 (17.6)
Child school grade^b^	
Mean (SD)	3.4 (2)
Min–Max	0–8
Years of IA behavior^b^	
Mean (SD)	4.9 (2.5)
Min–Max	0.75–11.67
Years since first consult^b^	
Mean (SD)	9.7 (3.5)
Min–Max	0.25–14.67
Type of doctor consulted, *n* (%)	
Primary care physician	38 (36.9)
Neurologist	10 (9.7)
Psychiatrist/psychologist	55 (53.4)
Child physical health, *n* (%)^d^	
Excellent	41 (39.8)
Very good	33 (32.0)
Good	23 (22.3)
Fair	4 (3.9)
Poor	2 (1.9)
Child mental health, *n* (%)^d^	
Excellent	7 (6.8)
Very good	18 (17.5)
Good	38 (36.9)
Fair	31 (30.1)
Poor	9 (8.7)

^a^ *N* represents the total subject sample.

^b^ Variables do not have a finite number of discrete response categories and were summarized with means, SDs, and minimum and maximum values rather than *n* (%).

^c^ One subject did not report race.

^d^ The child's physical and mental health were scored by the parent/caregiver using a provided questionnaire on which the parent/caregiver was asked to rate the child's health as “excellent, very good, good, fair, or poor.”

IA, impulsive aggression; Max, maximum; Min, minimum; SD, standard deviation.

### Sample composition

A total of 2341 IA diaries were collected, consisting of 2002 completed entries and 339 nonreports during the observation period. Nonreports were defined as entries in which no aggressive behaviors necessitating an IA diary entry occurred (as opposed to missing data). The mean number of IA diaries completed per subject was 15.1 (standard deviation [SD]: 13.5; range: 1–72 diaries), with completion on ∼10 days out of the 14-day assessment period (72.3%). Because the IA diary is episodic, participants could complete multiple diaries in a single day, as needed. The mean number of IA diaries completed per day was 1.4 (range: 0.14–5.14). Seventeen subjects completed 23 diaries outside the 14-day observation period and beyond the completion and recall periods of external validators, including R-MOAS and NCBRF. Considering this, a total of 1979 IA diaries were completed within an eligible time interval for analyses involving the R-MOAS or NCBRF.

### Psychometric validation and exploratory factor analysis

Psychometric validation revealed that of the 30 initially evaluated items (Supplementary Table S1), 15 (50.0%) were not reported at sufficient frequency for analysis (weighted prevalence rate <1%). These items were eliminated from the IA diary, based on item endorsement rates, inter-item correlations, and unconditional odds of item endorsement, including: teasing (removed due to an inverse relationship to IA detected through IRT modeling despite a prevalence score of 8.2), spitting, biting, using weapons, ripping, breaking, vandalizing, destroying, fire setting, hitting animals, kicking animals, severe injury to animals, kicking self, severe injury to self, or severe injury to others.

**FIG. 2.**
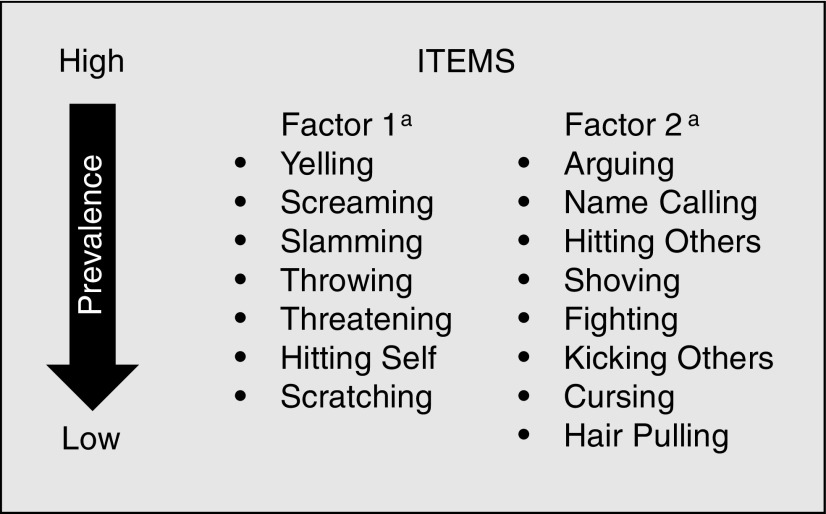
Item composition of the final IA diary. ^a^Factor categories based on how items performed and were grouped in IRT model. IA, impulsive aggression; IRT, item response theory.

Three items with weighted prevalence rates ≤1% (scratching, hair pulling, hitting self) were retained, as content validity was deemed high by clinical experts, and previous qualitative research suggested that these behaviors were more common than found in this study. The remaining 12 behavior items had weighted prevalence rates of 2.7%–73.6%. Thus, the final 15 items/behaviors were yelling, screaming, arguing, cursing, name calling, threatening, scratching, shoving, hair pulling, fighting, throwing, slamming, hitting self, hitting others, and kicking others (Fig. 2).

Exploratory factor analysis (EFA) and IRT modeling confirmed the existence of a general domain of IA behavior frequency with two subdomains in the IA diary (Fig. 2) and yielded a single total behavioral frequency score (TBFS). The first subdomain (7/15 items) was characterized by aggression items not always directed at human targets, including: yelling, screaming, threatening, scratching, throwing, slamming, and hitting self. The second subdomain (8/15 items) was characterized by more severe aggressive behaviors toward others, including: arguing, cursing, name calling, shoving, hair pulling, fighting, hitting others, and kicking others. The correlation between factors was *r* = 0.23.

While those items with an apparent higher severity tended to correlate with many other items in the diary, items with an apparent lower severity tended to correlate with fewer items in the diary. These associative features illustrate how items reflecting high-severity manifestations of aggression are more clearly associated with IA (Bland and Altman 1997).

### IRT model

Three alternative IRT models were explored: a one-parameter logistic (PL)/Rasch bifactor model, a two-factor 2PL IRT model adopting the EFA factor structure, and a 2PL bifactor IRT model. Both bifactor models adopted the EFA factor structure for the subfactors. Model fit was best for the 2PL bifactor IRT model, which has the lowest chi square and root mean squared error of approximation and the highest comparative fit index and Tucker–Lewis index (Supplementary Table S3).

### Scoring

Unweighted scores (from the EFA) and weighted scores (derived from the IRT models) were estimated to facilitate validation testing of the IA diary and evaluate the relationship of measured behaviors to the IA construct (Robb et al. 2017). Both the weighted and unweighted scores had strong psychometric properties, and reliability and validity estimates for weighted and unweighted scores were within the rounding error of one another (Table 2). Considering the high correlation between weighted and unweighted scores (*r* = 0.95), and the strong correspondence in reliability and validity estimates between the two scores, it is reasonable to conclude that the unweighted score is a good approximation of the weighted score. For the study group, the unweighted week 1 scores, summing the reported behaviors in each diary, ranged from 0 to 13.55, and the mean ± SD was 3.40 ± 2.10.

**Table 2.** Analysis Results Summary

		*Weighted scores*		*Unweighted scores*	
*Analysis*	*Criterion*	*Estimate*	*Succeed?*	*Estimate*	*Succeed?*
Internal consistency	≥0.8	0.86	Yes	0.73	No^c^
TRT	≥0.7–0.8	0.80	Yes	0.77	Yes
Concurrent validity R-MOAS^a^	≥0.4	0.58	Yes	0.49	Yes
Concurrent validity R-MOAS^b^	≥0.4	0.63	Yes	0.62	Yes
Concurrent validity NCBRF-TIQ D-Total	≥0.4	0.44	Yes	0.41	Yes
Known groups validity R-MOAS^a^	Strong positive effect	0.7 SD higher	Yes	111% higher^d^	Yes
Known groups validity R-MOAS^b^	Strong positive effect	0.44 SD higher	Yes	45% higher^d^	Yes
Known groups validity NCBRF-TIQ D-Total	Strong positive effect	0.35 SD higher	Yes	51% higher^d^	Yes

^a^ Administered on day 7.

^b^ Administered on day 14.

^c^ Although alpha values from 0.7 to 0.8 are often regarded as satisfactory (Bland and Altman 1997), the more stringent cutoff of 0.8 was used here.

^d^ Rate of behavior frequency.

NCBRF-TIQ, Nisonger Child Behavior Rating Form-Typical IQ; R-MOAS, Retrospective-Modified Overt Aggression Scale; SD, standard deviation; TRT, test–retest reliability.

### Internal consistency reliability

The Kuder Richardson-20 estimate of internal consistency for the unweighted IA diary score was 0.73. The marginal reliability corresponding to the weighted diary scores was 0.86. As described in the Supplementary Data section, alpha values of 0.7–0.8 are often regarded as satisfactory, but the more stringent cutoff of 0.8 was selected *a priori* for the current study.

### TRT

TRT for unweighted TBFS was based on Spearman rank correlations and was *r* = 0.77 for the total sample and *r* = 0.76 when conditioned on CGIC (no change). For weighted scores, TRT for the TBFS was 0.80, based on the ICC. For weighted scores, the ICC estimate and Pearson correlation demonstrated nearly perfect agreement. The unconditional TRT estimate was *r* = 0.80, while the TRT estimate conditioned on CGIC no-change status was slightly attenuated (*r* = 0.79) but nearly identical after rounding (*r* = 0.80).

### Concurrent validity

R-MOAS total score was used to calculate concurrent validity of TBFS, which ranged from *r* = 0.49 to *r* = 0.62. During week 1, 91 subjects completed R-MOAS and IA diaries in the R-MOAS recall period, generating a total score ranging from 4 to 137 (mean, 46.9 ± 27.6). For R-MOAS total scores, week 1 correlations with weighted and unweighted scores were *r* = 0.58 and *r* = 0.49, respectively. During week 2, 101 subjects completed the R-MOAS and IA diaries within the R-MOAS recall period, generating a total score ranging from 0 to 165 (mean, 42.4 ± 30.3). In week 2, the correlations between the R-MOAS and weighted and unweighted IA scores were *r* = 0.63 and *r* = 0.62, respectively.

In week 2, 101 subjects also completed the NCBRF-TIQ, generating a D-Total score ranging from 4 to 61 (mean, 34.6 ± 13.3). Scores ≥48 in the NCBRF-TIQ D-Total distribution corresponded to the 85th percentile; therefore, scores of 48 or greater defined the NCBRF-TIQ D-Total severity group. For NCBRF-TIQ D-Total scores, the correlations with weighted and unweighted IA scores that averaged across the entire study period were *r* = 0.44 and *r* = 0.41, respectively.

### Known groups validity

In week 1, unweighted IA scores predicted by R-MOAS groups had a rate of behavior reporting of 111% (rate ratio [RR] = 2.11, 95% confidence interval [CI]: 1.61–2.77; *p* < 0.0001) greater in the R-MOAS high-severity group versus the R-MOAS low-severity group. For weighted IA scores predicted by R-MOAS groups, scores in the first week of observation were 0.70 (95% CI: 0.41–0.99; *p* < 0.0001) SDs higher in the R-MOAS high-severity group versus the R-MOAS low-severity group. In the second week, unweighted IA scores predicted by R-MOAS groups had a rate of behavior reporting of 45% (RR = 1.45, 95% CI: 1.16–1.80; *p* = 0.0009) higher in the R-MOAS high-severity group versus the low-severity group. For weighted scores predicted by the R-MOAS groups, scores in the second week were 0.44 (95% CI: 0.19–0.69, *p* = 0.0005) SDs higher in the R-MOAS high-severity group versus the R-MOAS low-severity group.

When the same scores were predicted by the NCBRF-TIQ D-Total severity groups, the results were similar to the known groups conducted with the R-MOAS. For unweighted IA scores across the entire observation period, the rate of behavior reporting in diaries was 51% (RR = 1.51, 95% CI: 1.18–1.92; *p* = 0.0012) higher in the D-Total high-severity group versus the D-Total low-severity group. For weighted IA scores, the rate of behavior reporting was 0.35 (95% CI: 0.16–0.55; *p* = 0.001) SDs higher in the D-Total high-severity group versus the D-Total low-severity group.

## Discussion

IA behavior is a serious clinical concern for patients and families affected by ADHD. The lack of a comprehensive assessment tool has remained a roadblock in understanding, accurately identifying, and monitoring IA. The development of an accessible, robust IA measurement tool to capture behavior frequency will permit clinical researchers and physicians to accurately assess IA behaviors and monitor changes over time. These analyses indicate that the 15-item IA diary is a valid reliable tool for assessment of IA behaviors in children with ADHD (Fig. 2). Based on the psychometric tests conducted, all but one test exceeded the prespecified threshold values for test success (Table 2).

This study indicates that the established psychometric properties and scoring can effectively characterize IA across a spectrum of verbal and physical behaviors. Real-time or same-day measurement tools help track symptoms without the burden of recall bias on the day of an appointment, assisting families and clinicians in accurately tracking behavioral patterns over time. The validation data presented here utilized empirical model-based evidence to guide development of the IA diary, culminating in a core set of 15 behaviors that were strongly associated with and effective at detecting IA.

Of the initial item pool of 30 behaviors developed through saturation analysis of qualitative interviews with children and caregiver pairs, 15 consistently occurred with sufficient prevalence in children with ADHD and IA. It should be noted that the prevalence of many of the eliminated IA behaviors was not known *a priori*. Many of the behaviors for which our analyses revealed insufficient prevalence had been bundled together within the R-MOAS. While the R-MOAS addresses behavior prevalence, it also includes rarely occurring severe behaviors and has an arbitrary weighting system, creating difficulties differentiating IA from other forms of aggression (Jensen et al. 2007). The novel IA diary's 15-item subset contains psychometrically validated behaviors whose unique contributions to defining IA are clearly and empirically supported.

Furthermore, when comparing the IA diary to existing scales such as the R-MOAS and NCBRF, concurrent validity correlations would be expected to be modest, as these metrics measure IA symptomatology in different ways. The IA diary collects information daily, requiring only very short recall from the observer to recount aggressive episodes. Importantly, NCBRF utilizes a 1-month recall period, while the R-MOAS has a 1-week recall period, which correlates better with the contemporaneous IA diary. Thus, while every IA diary aligned within the R-MOAS recall period, diaries collected over the entire 2-week study overlapped with only 50% of the 1-month NCBRF recall period.

These differences, and the missing information for events occurring in the 2 weeks preceding study initiation, may have contributed to the lower estimates for the NCBRF than for the R-MOAS. Although estimates for the unweighted versus weighted scores were nearly identical in most cases, correlations were higher between weighted IA diary scores and the R-MOAS and NCBRF than they were for unweighted scores.

One potential limitation of this study is that due to low sample size, possibly relevant behaviors occurring at low prevalence were dropped from the diary. However, all items with low prevalence were evaluated by clinical experts and if deemed clinically appropriate were retained in the IA diary (i.e., scratching, hair pulling, and hitting self). Low prevalence items may also be a result of the study's sample demographics. As only 29% of the study population was female, it remains possible that potential gender-specific behaviors were not adequately captured. However, given that the male:female ratio in this study (∼2:1) is representative of the prevalence of ADHD in the U.S. population and that the prevalence of aggressive behaviors has been reported to be higher in males with ADHD, it is likely that the IA diary sufficiently captures the most prevalent behaviors observed in the clinic (Rucklidge 2010; Children and Adults with Attention-Deficit/Hyperactivity Disorder [CHADD] 2017).

It should also be noted that the IA diary has only been validated in children of ages 6–12 who have ADHD and signs of aggression, and results from this study should not be extrapolated to other populations or conditions. Moreover, the IA diary is an observer-reported tool, meaning that some IA behaviors may not be recorded if the behavior occurs in the absence of or is omitted by the observer. As it is suspected that children may be less likely to accurately self-report negative behaviors, observer-based reporting is necessary.

## Conclusions

This novel instrument demonstrates a strong model fit and psychometric properties, making the 15-item IA diary a clinically useful tool for characterizing symptomatic IA patients in real time. This study supports the validity and reliability of the IA diary in children with ADHD and corroborates the rationale for using this unique metric in future studies. These results also provide basic epidemiologic data on the prevalence and rarity of behaviors assumed to be representative of IA, which were not empirically assessed previously.

## Clinical Significance

The validity of the IA diary is supported by an additional qualitative research study in adolescents and ongoing Phase 3 trials to determine if IA improves with SPN-810 (extended-release molindone) treatment in children being treated for ADHD (Robb et al. 2019). As the use of the IA diary as a clinical outcome measure in this Phase 3 study is ongoing, it remains to be determined whether the diary is sensitive to changes over time or in response to treatment. As this tool is incorporated into further studies, we will gain more insight about its utility in clinical practice and how it may be used to assess children with ADHD and IA.

## Supplementary Material

Supplemental data
