# Supplementary material for: A Novel Assessment Tool for Impulsive Aggression in Children with Attention-Deficit/Hyperactivity Disorder
Source: J Child Adolesc Psychopharmacol. 2019 Oct 7;29(8):592–8. doi: 10.1089/cap.2019.0035 (PMC6786341; doi:10.1089/cap.2019.0035)
Supplement: Supplemental data [file Supp_Data.pdf]

Supplementary Data

Provisional Impulsive Aggression Diary

Items in the provisional diary were developed through a qualitative research study by conducting semistructured qualitative interviews with 23 pairs of caregivers and children with attention-deficit/hyperactivity disorder (aged 6–12 years). Following these interviews, saturation analysis (i.e., the point at which no new themes or information emerged) and content validity were established for 30 single-item actions (behaviors), generating the provisional impulsive aggression (IA) diary (Supplementary Table S1).

Clinical Setting and Inclusion/Exclusion Criteria

Subjects were recruited from five U.S. clinical sites, specifically led by the following investigators/clinicians: Robert Findling, MD, at the Hugo Moser Research Institute at Kennedy Krieger, Baltimore, MD; Jill Hollway, PhD, at The Ohio State University, Columbus, OH; Richard Litov, PhD, at Pedia Research, Owensboro, KY; Conrado Beckles, PhD, at Meridian Research (formerly known as the Florida Clinical Research Center), Maitland, FL; and Joanne Northcutt, PhD, also at Meridian Research, Maitland, FL.

Enrollment required a willingness to complete the electronic IA diary for 14 days, the paper Retrospective-Modified Overt Aggression Scale twice during the study, and the paper Nisonger Child Behavior Rating Form-Typical IQ and the Caregiver Global Impression of Change once during the study. Among the study's exclusion criteria were a current or lifetime diagnosis of major depressive disorder, bipolar disorder, personality disorder, autism spectrum disorder, or psychosis not otherwise specified. The presence of any of these disorders was determined using parent/caregiver questionnaire responses.

Psychometric Analyses: Model Fit Indices and Standard Algorithms

Model fit indices generated using exploratory factor analysis (EFA) were used to determine dimensionality and factor compo-

Supplementary Table S1. Full Set of Behaviors Included in the Provisional Impulsive Aggression Diary

Provisional diary behaviors	
1. Yelling	16. Slamming
2. Screaming	17. Ripping
3. Arguing	18. Breaking
4. Cursing	19. Vandalizing
5. Name calling	20. Destroying
6. Teasing	21. Fire setting
7. Threatening	22. Hitting self
8. Spitting	23. Hitting others
9. Biting	24. Hitting animal
10. Scratching	25. Kicking self
11. Shoving	26. Kicking others
12. Hair pulling	27. Kicking animal
13. Fighting	28. Severe injury self
14. Weapons	29. Severe injury others
15. Throwing	30. Severe injury animal

Supplementary Table S2. Reporter Demographics

Variable	*Estimates* (N = *103)*[a]
Reporter sex, *n* (%)	
Male	6 (5.8)
Female	97 (94.2)
Reporter age[b]	
Mean (SD)	36.6 (8.8)
Min–Max	24–67
Reporter education, *n* (%)	
Less than high school	1 (1.0)
Some high school	13 (12.6)
High school diploma/GED	23 (22.3)
Some college	37 (35.9)
BA/BS	16 (15.5)
MA/MS/PhD	8 (7.8)
Vocational	5 (4.9)
Reporter employment, *n* (%)	
FT/PT	54 (52.4)
Looking	9 (8.7)
FT home	25 (24.3)
FT student	4 (3.9)
Disability	7 (6.8)
Retired	3 (2.9)
Prefer not to answer	1 (1)
Living arrangements of reporter, *n* (%)	
Living alone	27 (26.2)
Living w/partner	23 (22.3)
Living w/partner and children	34 (33)
Living w/parents	17 (16.5)
Living w/friends	2 (1.9)
Marital status of reporter, *n* (%)	
Single	42 (40.8)
Married	49 (47.6)
Divorced	11 (10.7)
Widowed	1 (1.0)
Other children in the home, *n* (%)	
No other children at home	9 (8.7)
Yes, other children at home	94 (91.3)
Number of children[b]	
Mean (SD)	3.4 (1.6)
Min–Max	1–8
Number of children w/ADHD[b]	
Mean (SD)	2.1 (1.2)
Min–Max	0–5
Annual household income of reporter, *n* (%)	
<$25,000	52 (50.5)
$25,000–$49,999	28 (27.2)
$50,000–$74,999	7 (6.8)
$75,000–$99,999	5 (4.9)
≥$100,000	7 (6.8)
Prefer not to answer	4 (3.9)

[a]*N* represents the total subject sample.

[b]Variables do not have a finite number of discrete response categories and were summarized with means, standard deviations, and minimum and maximum values rather than *n* (%).

ADHD, attention-deficit/hyperactivity disorder; FT, full-time; GED, general educational development; Max, maximum; Min, minimum; PT, part-time; SD, standard deviation.

Supplementary Table S3. Model Fit, Item Response Theory Models

Model	p (χ^2)	*RMSEA*	*LCL*	*UCL*	*CFI*	*TLI*
Two factor 2PL	0.196	0.035	0	0.066	0.935	0.924
Bi-factor 2PL	0.661	0	0	0.047	1	1.045
Bi-factor Rasch	0.037	0.051	0.013	0.076	0.845	0.841

The 2PL estimates both a threshold and log odds ratio (referred to as a discrimination parameter in IRT parlance) for each item. The Rasch model, also known as the 1PL, is identical to the 2PL except that it constrains the log odds ratio to equality across items and freely estimates the threshold for each item. In the χ^2 statistic, the comparator model is a saturated model, and a nonsignificant *p*-value indicates good model fit. The RMSEA quantifies model fit per degree of freedom, in which a good fit is indicated by values of 0.05 or lower. The statistic is bounded at 0, and confidence limits with an LCL of 0 and UCL <0.05 indicate excellent fit. The CFI and TLI are bounded between 0 and 1, although in rare instances the TLI can exceed 1. Values above 0.9 indicate good fit.

2PL, two-parameter logistic; CFI, comparative fit index; IRT, item response theory; LCL, lower confidence limit; RMSEA, root mean squared error of approximation; TLI, Tucker–Lewis Index; UCL, upper confidence limit.

sition of the scale, wherein factor structure was estimated by generalized linear EFA modeling, and oblique varimax rotated loading patterns were used to determine IA diary dimensionality. Item response theory (IRT) analysis was estimated using the two-parameter logistic (2PL) IRT model, with item quality characterized using slopes and thresholds. Model fit indices were used to select the appropriate IRT model for the IA diary. Two models were considered for the binary IA diary items: the 2PL and the Rasch or one-parameter logistic (1PL) IRT model. These models differ only in the number of slopes estimated (2PL estimates one slope/item; Rasch/1PL estimates no slope/item), and the 2PL was retained as the best fit.

Standard algorithms of the Bock and Mislevy (1982) empirical Bayes score method were used to generate weighted scores. Unweighted scores were generated as the sum of the binary reported behaviors in each participant's IA diary, compared to weighted *z*-scores. Internal consistency reliability was estimated by the Kuder–Richardson Formula 20 (KR-20) for unweighted scores and by IRT marginal reliability for weighted scores. KR-20 is the analog of Cronbach's alpha for binary items, and marginal reliability is the IRT model-based analog for Cronbach's alpha. Although alpha values of 0.7–0.8 are regarded as satisfactory (Bland and Altman 1997), the more stringent cutoff of 0.8 was chosen *a priori*.

Supplementary References

Bland JM, Altman DG: Cronbach's alpha. BMJ 314:572, 1997.
Bock R, Mislevy RJ: Adaptive EAP estimation of ability in a microcomputer environment. Appl Psychol Meas 6:431–444, 1982.
